# Production of functionalized oligo‐isoprenoids by enzymatic cleavage of rubber

**DOI:** 10.1111/1751-7915.12748

**Published:** 2017-07-11

**Authors:** Wolf Röther, Jakob Birke, Stephanie Grond, Jose Manuel Beltran, Dieter Jendrossek

**Affiliations:** ^1^ Institute of Microbiology University of Stuttgart Stuttgart Germany; ^2^ Institute of Organic Chemistry Eberhard Karls Universität Tübingen Tübingen Germany

## Abstract

In this study, we show the proof of concept for the production of defined oligo‐isoprenoids with terminal functional groups that can be used as starting materials for various purposes including the synthesis of isoprenoid‐based plastics. To this end, we used three types of rubber oxygenases for the enzymatic cleavage of rubber [poly(*cis*‐1,4‐isoprene)]. Two enzymes, rubber oxygenase RoxA_X__sp_ and rubber oxygenase RoxB_X__sp_, originate from *Xanthomonas* sp. 35Y; the third rubber oxygenase, latex‐clearing protein (Lcp_K30_), is derived from Gram‐positive rubber degraders such as *Streptomyces* sp. K30. Emulsions of polyisoprene (latex) were treated with RoxA_X__sp_, RoxB_X__sp_, Lcp_K30_ or with combinations of the three proteins. The cleavage products were purified by solvent extraction and FPLC separation. All products had the same general structure with terminal functions (CHO‐CH_2_‐ and ‐CH_2_‐COCH_3_) but differed in the number of intact isoprene units in between. The composition and *m/z* values of oligo‐isoprenoid products were determined by HPLC‐MS analysis. Our results provide a method for the preparation of reactive oligo‐isoprenoids that can likely be used to convert polyisoprene latex or rubber waste materials into value‐added molecules, biofuels, polyurethanes or other polymers.

## Introduction

Natural rubber has been produced in huge amounts for more than a century by cultivating the rubber tree (*Hevea brasiliensis*), and the material is used for a variety of applications, as an example for the production of rubbers, tyres, sealings, latex gloves and many other items. The main component of rubber is the hydrocarbon poly(*cis*‐1,4‐isoprene). For most of today's applications of rubber, an important material property is the molecular weight of the polymer that – when high – gives rise to superior material properties that are necessary for example for the production of tyres. However, no attention has been given so far to the use of rubber for the biotechnological preparation of low molecular fine chemicals (Förster‐Fromme and Jendrossek, 2010; Kamm, 2014; Akhlaghi *et al*., 2015; Schrader and Bohlmann, 2015). In this contribution, we describe the proof of concept for the use of rubber oxygenases to cleave polyisoprene‐containing (waste) materials to low molecular products and to produce functionalized oligo‐isoprenoids with defined structure. The generated products can be used either directly as biofuels or value‐added materials which can be obtained by conversion of oligo‐isoprenoids to new products such as polyurethanes and related isoprene‐containing polymers.

**Figure 1:**
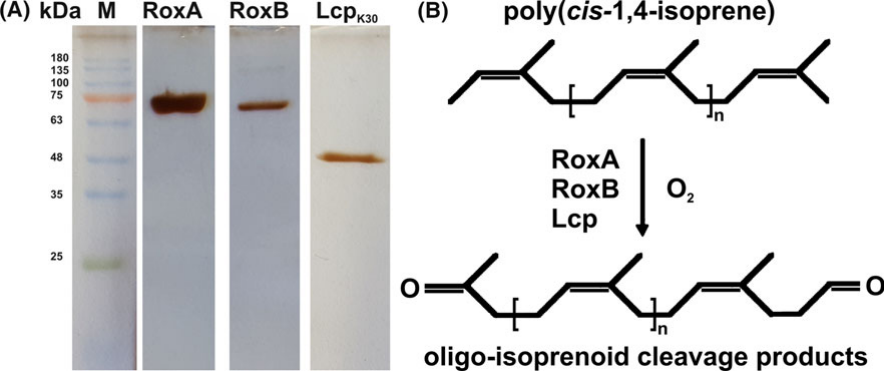
SDS‐PAGE of purified rubber oxygenases. RoxA_X__sp_ and RoxB_X__sp_ were purified from filter‐concentrated supernatants of L‐rhamnose/LB‐medium‐grown *∆roxA Xanthomonas* sp. 35Y cells with genome‐integrated *rox* *A*_X__sp_ or *rox* *B*_X__sp_ gene respectively. Lcp_K30_ was purified from soluble French‐press extracts of L‐rhamnose/LB‐medium‐grown *E. coli* (p4782.1::*strep‐lcp*_K30_) via Strep‐Tactin HC gravity flow affinity chromatography. A. Purified proteins were separated by SDS‐PAGE and stained with silver. A molecular mass standard (M) with kDa values indicated is shown. B. Oxidative cleavage of rubber. Poly(*cis*‐1,4‐isoprene) (100 < *n* < ≈ 10 000) is oxidatively cleaved by rubber oxygenases to oligo‐isoprenoids with terminal keto‐ and aldehyde groups. The methanol‐soluble products differ in the number of intact isoprene units (*n*) with 1 ≤ *n* < ≈ 12.

Only two major types of rubber‐cleaving enzymes have been described so far. One is the rubber oxygenase RoxA that was first isolated from *Xanthomonas* sp. 35Y (Tsuchii and Takeda, 1990; Braaz *et al*., 2004) and has been found only in Gram‐negative rubber‐degrading bacteria (Birke *et al*., 2013). The genome sequence of *Xanthomonas* sp. 35Y has been determined (Sharma, V., Siedenburg, G., Birke, J., Mobeen, F., Jendrossek, D., Srivastava, T.P. unpubl. data). RoxA of *Xanthomonas* sp. 35Y (RoxA_Xsp_) is a *c*‐type dihaem dioxygenase (≈70 kDa, Fig. 1A) and cleaves poly(*cis*‐1,4‐isoprene) into 2‐oxo‐4,8‐dimethyl‐trideca‐4,8‐diene‐1‐al (ODTD), a C_15_ compound with a terminal keto and aldehyde group as the main product (Fig. 1B) (Braaz *et al*., 2005; Schmitt *et al*., 2010). The structure of RoxA_Xsp_ has been solved (Seidel *et al*., 2013), and molecular insights in the active site of RoxA_Xsp_ as well as the cleavage mechanism have been obtained by the construction and biochemical characterization of RoxA_Xsp_ muteins (Birke *et al*., 2012).

**Figure 2:**
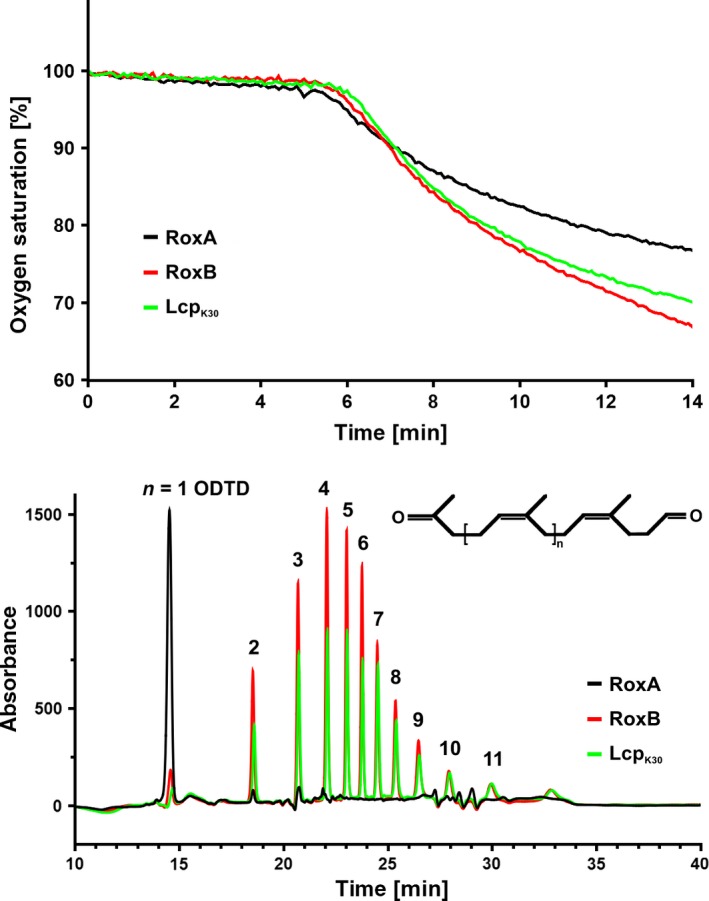
Activities and product analysis of rubber oxygenases. Activities of purified rubber oxygenases (Lcp_K30,_ RoxA_X__sp_ and RoxB_X__sp_) were determined by following the consumption of dissolved oxygen at 37°C in a Oxy4 V2 apparatus, Presens, Regensburg, Germany, as described recently (Röther *et al*., 2017) (top). 4 μg each of Lcp_K30,_ RoxA_X__sp_ or RoxB_X__sp_ was added to 1 ml of an emulsion of polyisoprene latex in potassium phosphate buffer (100 mM, pH 7) at ≈5.5 min. The initial slopes correspond to specific activities of 6.2, 2.6, 6.4 U mg^−1^ for Lcp_K30,_ RoxA_X__sp_ or RoxB_X__sp_ respectively. One unit corresponds to the consumption of one molecule of dioxygen per minute. The products of polyisoprene cleavage were determined by HPLC‐based analysis of the ethylacetate‐extracted cleavage products (bottom). For Lcp_K30_ and RoxB_X__sp,_ a typical pattern of oligo‐isoprenoids varying in the number of subunits (*n* = 2–11) was observed. For RoxA, 12‐oxo‐4,8‐dimethyltrideca‐4,8‐diene‐1‐al (ODTD,* n* = 1) was detected as the major cleavage product.

The second type of rubber oxygenase is a protein designated as latex‐clearing protein (Lcp) (Rose *et al*., 2005; Hiessl *et al*., 2012; Yikmis and Steinbüchel, 2012). Lcps (≈40 kDa, Fig. 1A) are widespread in or even specific for Gram‐positive rubber‐degrading bacteria, such as *Streptomyces* sp. K30 (Lcp_K30_) (Rose *et al*., 2005), *Gordonia polyisoprenivorans, Gordonia westfalica* (Arenskötter *et al*., 2001; Bröker *et al*., 2008), and were recently isolated from *Gordonia polyisoprenivorans* VH2 (Hiessl *et al*., 2014) *Streptomyces sp*. K30 (Birke *et al*., 2015; Röther *et al*., 2016) and from *Rhodococcus rhodochrous* RPK1 (Watcharakul *et al*., 2016). The amino acid sequences of RoxAs and Lcps are not related although both enzymes catalyse the oxidative cleavage of the double bonds in poly(*cis*‐1,4‐isoprene) and both cleave polyisoprene to products with terminal keto and aldehyde groups (Fig. 1B). In contrast to RoxAs that cleave rubber to only one major end‐product (ODTD), Lcps produce a mixture of oligo‐isoprenoids (C_20_, C_25_, C_30_ and higher oligo‐isoprenoids, Fig. 2) (Ibrahim *et al*., 2006; Birke and Jendrossek, 2014). Lcps are *b*‐type cytochromes and share a common domain of unknown function (DUF2236) (Hiessl *et al*., 2014; Birke *et al*., 2015). Recently, the importance of several strictly conserved residues within the DUF2236 domain for stability and activity was determined (Röther *et al*., 2016).

Very recently, a third type of rubber oxygenase, RoxB, was discovered (Birke *et al*., 2017). The coding sequence is provided under the accession No KY 498024. RoxB_Xsp_ was identified as a RoxA_Xsp_ homologue in *Xanthomonas* sp. 35Y and shared some properties with RoxAs: RoxB_Xsp_ is also a *c*‐type dihaem protein with an apparent molecular weight of around 70 kDa), but it has only a low sequence similarity to RoxA_Xsp_ (38%). However, RoxB_Xsp_ differs from RoxAs in cleaving polyisoprene to a mixture of oligo‐isoprenoids (C_20_, C_25_, C_30_ and higher oligo‐isoprenoids, Fig. 2B). This has previously been described only for Lcps. Therefore, RoxB_Xsp_ combines properties of RoxAs and Lcps (Birke *et al*., 2017). RoxB is related in amino acid sequence to the *latA* gene product of *Rhizobacter gummiphilus* (83%) (Kasai *et al*., 2017). The *latA* gene was recently discovered to code for a protein that is responsible for the cleavage of polyisoprene in *R. gummiphilus*. However, no information on the properties of the expressed LatA protein is yet available.

### Recombinant overexpression of rubber oxygenases

Despite the fact that all so far described rubber oxygenases must be post‐translationary modified to incorporate the haem cofactor, overexpression of highly active rubber oxygenases is surprisingly easy: RoxA_Xsp_ can be expressed extracellularly in quantities of ≈15 mg l^−1^ from recombinant *Xanthomonas* sp. 35Y strains which harbour a *roxA*_Xsp_ gene on the chromosome under the control of an rhamnose‐inducible promoter (Hambsch *et al*., 2010; Birke *et al*., 2012). We assume that the amount of produced rubber oxygenase can be increased by a combination of medium optimization, inducer concentration and the time point of addition and harvest. Lcps have been successfully overexpressed intracellularly in recombinant *E. coli* using conventional induction by rhamnose (Birke *et al*., 2015; Watcharakul *et al*., 2016) or via autoinduction (Andler and Steinbüchel, 2017). Secretion of the mature Lcps via the TAT secretion pathway in *E. coli* (Yikmis *et al*., 2008) or *Bacillus subtilis* (van Dijl and Hecker, 2013) should be possible. However, the secretion pathways used for RoxA and RoxB proteins have not yet been identified. If pure proteins are necessary, tagged versions of Lcps can be purified in high yields using a one step affinity chromatography (≈ 15 mg Lcp_K30_ l^−1^ culture for Strep‐tagged Lcp). The tag also offers the opportunity for enzyme immobilization. Furthermore, over‐production of haem containing rubber oxygenases might be limited by the intracellular availability of the cofactor. An increase in the efficiency of haem biosynthesis, e g., by the expression of gamma‐aminolevulinic acid synthase and gamma‐aminolevulinic acid dehydratase could be used to overcome this limitation (Doss and Philipp‐Dormston, 1975).

### Purification of rubber oxygenases

We purified each one representative of the three types of rubber oxygenase (RoxA_Xsp_, RoxB_Xsp_ and Lcp_K30_, Fig. 1) and used the purified proteins alone or in combination for the production of oligo‐isoprenoids from polyisoprene latex. Produced oligo‐isoprenoids were purified by HPLC and FPLC, and the identity of the isolated products was confirmed by ESI‐MS analysis.

Untagged RoxA_Xsp_ and RoxB_Xsp_ were purified from the culture fluid of recombinant *∆roxA Xanthomonas* sp. 35Y strains which harboured either the *roxA*_Xsp_ or the *roxB*_Xsp_ gene integrated into the chromosome under the control of an L‐rhamnose‐inducible promoter using a two‐step purification procedure as described recently (Birke *et al*., 2012, 2017). Lcp_K30_ was expressed intracellularly in form of an N‐terminal Strep‐tagged protein and was purified from recombinant *E. coli* as described previously (Röther *et al*., 2016). Fig. 1A shows that all three proteins were of high purity and activity determinations confirmed high specific activities of 2.6 U mg^−1^ (RoxA_Xsp_), 6.2 U mg^−1^ (Lcp_K30_) and 6.4 U mg^−1^ (RoxB_Xsp_) at 37°C for the three purified rubber oxygenases (Fig. 2 top). HPLC analysis of the solvent‐extracted products confirmed the cleavage of polyisoprene to ODTD (C_15_ oligo‐isoprenoid) as major product by RoxA_Xsp_ and the formation of a mixture of C_20_ and higher oligo‐isoprenoids in case of RoxB_Xsp_ and Lcp_K30_ (Fig. 2 bottom). ODTD was present only in minor amounts in the products obtained from RoxB_Xsp_ and Lcp_K30_.

The finding of only one cleavage product (C_15_ oligo‐isoprenoid ODTD) for the RoxA_Xsp‐_catalysed reaction and the identification of multiple cleavage products (C_20_ and higher oligo‐isoprenoids) in case of the RoxB_Xsp_‐ or Lcp_K30_‐cleaved polyisoprene suggested that RoxA_Xsp_ on the one side and RoxB_Xsp_ and Lcp_K30_ on the other side employ different cleavage mechanisms. We assume that RoxA_Xsp_ has a ‘molecular ruler’ and uses an *exo*‐type mechanism to cleave the polyisoprene chain (Seidel *et al*., 2013). This explains the formation of only one main cleavage product of a defined length (ODTD). In contrast, in case of RoxB_Xsp_ and Lcp_K30,_ the formation of multiple products of different length suggests that these rubber oxygenases do not have such a molecular ruler and cleave the polyisoprene chain randomly in an *endo‐*type mechanism resulting in the observed mixture of oligo‐isoprenoids of different lengths.

### Synergistic effect of RoxB and of Lcp on polyisoprene cleavage by RoxA

**Figure 3:**
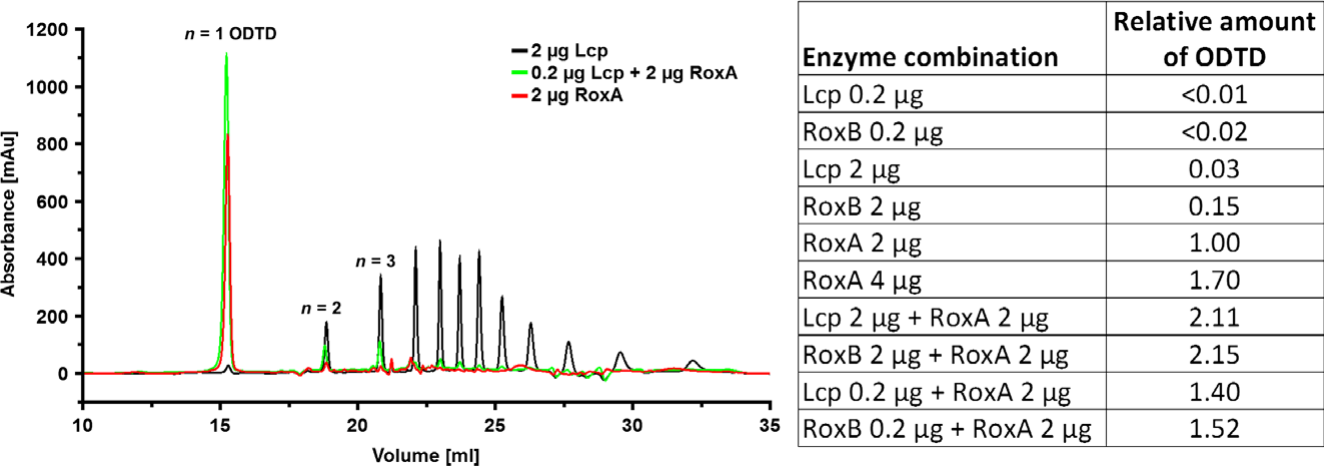
Synergistic effect during rubber cleavage. Polyisoprene latex was cleaved by different amounts and combinations of rubber oxygenases as indicated (left). The amounts of produced ODTD (Table on the right) were determined from the ODTD areas in HPLC chromatograms (exemplary shown in the image on the left). The ODTD‐specific area obtained for 2 μg of RoxA_X__sp_ was set as 1.0. The addition of only 0.2 μg Lcp increased ODTD formation by 2 μg of RoxA_X__sp_ by a factor of 1.4 and only trace amounts of higher oligo‐isoprenoids (*n* = 2 and 3; *n* indicates the number of intact isoprene units, see structure shown in Fig. 1B) were determined. A similar effect with 1.5‐fold higher amount of produced ODTD was observed for a combination of 2 μg RoxA_X__sp_ and 0.2 μg RoxB_X__sp_.

**Figure 4:**
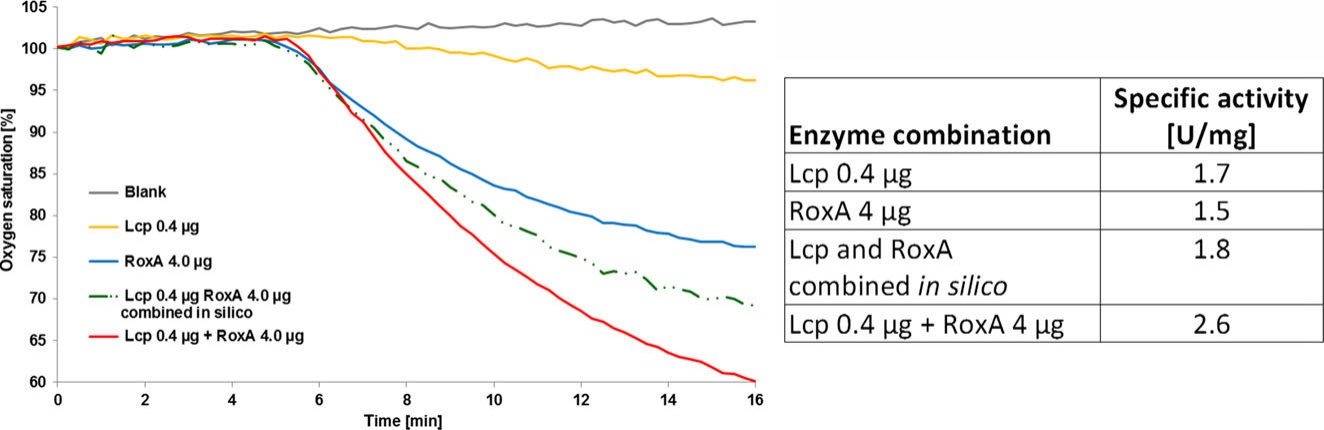
Synergistic effect of the presence of Lcp_K30_ on the specific activity of RoxA_X__sp_. The oxygen consumption rates of 0.4 μg Lcp_K30_, of 4 μg of RoxA_X__sp_ and of a mixture of 0.4 μg Lcp_K30_ and of 4 μg of RoxA_X__sp_ were recorded. The values for 0.4 μg Lcp_K30_ and 4 μg RoxA_Xsp_ were combined *in silico* and the slope of the resulting curve was calculated to determine a theoretical specific activity. Note that the specific activities of the reaction in the presence of both enzymes were 1.4‐fold higher (2.6 U mg^−1^) compared to the *in silico* combined values of the two reactions with the single enzymes (1.8 U mg^−1^).

The generation of oligo‐isoprenoids by *endo*‐cleavage of polyisoprene molecules (with RoxB_Xsp_ or Lcp_K30_) increases the number of free polyisoprene chains. A higher concentration of polyisoprenoid ends should enhance the efficiency of polyisoprene cleavage by rubber oxygenases with an endo‐type cleavage such as RoxA_Xsp._ We therefore determined whether the amount of ODTD produced by RoxA could be increased by the presence of trace amounts of RoxB_Xsp_ or Lcp_K30_. The presence of 0.2 μg ml^−1^ purified RoxB_Xsp_ or Lcp_K30_ in the assay mixture did not lead to the formation of substantial amounts of ODTD (factor being < 0.02 relative to 1.0 by 2 μg of RoxA_Xsp_, Fig. 3). However, when combined, 2 μg ml^−1^ RoxA_Xsp_ and 0.2 μg ml^−1^ purified RoxB_Xsp_ or Lcp_K30_ increased the amount of produced ODTD by a factor of 1.4 or 1.5, respectively, in comparison with the values obtained with 2 μg RoxA_Xsp_ or Lcp_K30_ alone (Fig. 3). Furthermore, the synergistic effect was investigated with respect to a kinetic effect enhancing the speed of the cleavage reaction, representing a major factor to be considered upon industrial employment of the reaction. To this end, the oxygen consumption rates by Lcp_K30_ (0.4 μg) and RoxA_Xsp_ (4 μg) alone were determined, combined (added) *in silico* and were then compared to an experiment in which both enzymes were simultaneously present. As evident from Fig. 4, the simultaneous presence of low amounts of Lcp_K30_ increased the specific oxygen consumption by a factor of 1.4 (2.6 U mg^−1^) relative to the *in silico* combined oxygen consumption rates (1.8 U mg^−1^). These results also showed that the presence of terminal aldehyde and keto groups did not inhibit the cleavage of these oligo‐isoprenoids to ODTD by RoxA_Xsp_. Furthermore, the efficiency of rubber degradation was enhanced when each an *endo‐* and *exo*‐type rubber oxygenase were simultaneously present. These data provide a plausible explanation for the presence of the *roxA* and *roxB* gene in *Xanthomonas* sp. 35Y due to a synergistic effect; in the presence of both gene products, ODTD is the only observed cleavage product for the facilitated uptake into the cells and use as a source of carbon and energy.

### Separation and purification of oligo‐isoprenoids

**Figure 5:**
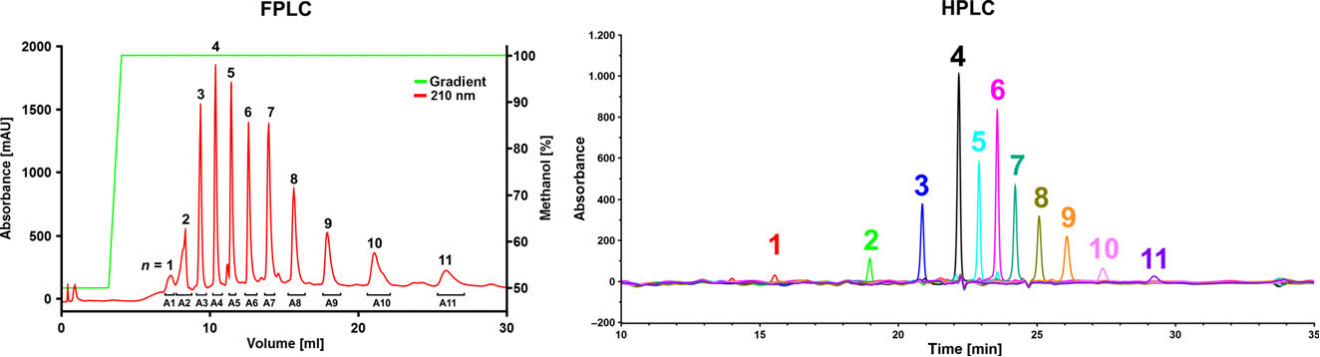
Separation of oligo‐isoprenoid mixtures by FPLC, HPLC and HPLC‐MS. 200 μl oligo‐isoprenoid solution in methanol (prepared by digestion of polyisoprene with Lcp_K30_ as described in the main text) was applied to a reversed‐phase FPLC column (Pep RPC HR 5/5, 1 ml bed volume) and separated by a step gradient from 50% water: methanol to 100% methanol (green line) (left image). Absorption at 210 nm (red line) was used to fractionate peaks representing different oligo‐isoprenoids (A1‐A11, corresponding to *n* = 1–11). Aliquots of each separately collected fraction (A1 to A11) were applied to analysis via HPLC. An overlay image consisting of all eleven HPLC chromatograms is shown on the right. The superposition of the chromatograms shows the high resolution power of the used FPLC column. The masses (*m/z* values) of each isolated compound were confirmed by HPLC‐ESI‐MS and are provided in Table 1.

As shown in Fig 2B, the cleavage of polyisoprene by RoxB_Xsp_ or by Lcp_K30_ yielded a mixture of oligo‐isoprenoids (C_20_ and higher oligo‐isoprenoids). For the application of these compounds as fine chemicals or as building blocks for (polymer) plastic synthesis in organic chemistry, the preparation of large amounts of pure oligo‐isoprenoids is preferable. To demonstrate the isolation of isoprenoids at a higher scale, we increased the volume of polyisoprene latex and replaced the HPLC‐based separation of oligo‐isoprenoids by an FPLC separation system because FPLC systems can be up‐scaled more easily than HPLC‐based separations. As a proof of principle, we treated 1 litre of 5% (wt/vol) polyisoprene latex in 100 mM potassium phosphate buffer, pH 7 with 4 mg of purified Lcp_K30_ and incubated the assay mixture for 24 h at room temperature while stirring at 200 rpm. The produced oligo‐isoprenoids were solvent‐extracted with 100 ml ethylacetate. The solvent was evaporated, and the products (≈100 mg) were dissolved in 5 ml methanol. 200 μl of the dissolved products was then applied to a PEP RPC HR5/5‐FPLC column that had been equilibrated with 50% methanol: water and eluted by the application of an increasing step gradient to 100% methanol at a constant flow rate of 1.5 ml min^−1^. Peaks were automatically fractioned (≈2 ml per peak) by monitoring the absorbance at 210 nm. As shown in Fig. 5 left, the same eleven individual peaks were identified that had been detected on the analytical HPLC column (Fig. 2). The compound of each of the separated peaks was collected individually, concentrated by evaporation and dissolved in 100 μl of methanol. When each of the isolated compounds was separately run on the analytical HPLC column, the successful isolation of each oligo‐isoprenoid was demonstrated by the appearance of one homogeneous peak (Fig. 5, right). The *m/z* values of the isolated oligo‐isoprenoids were determined by HPLC‐MS and were in agreement with the structural formulas and the theoretical values for the individual oligo‐isoprenoids (Table 1).

**Table 1:** Oligo‐isoprenoids produced by enzymatic cleavage of polyisoprene using purified rubber oxygenases

No of intact isoprene units [*n*]	Elemental formula	*m/z*	*m/z* [M+H]^+^	*m/z* [M+Na]^+^	*m/z* [M+Na+CH_3_OH]^+^	FPLC peak area [%]
1	C_15_H_24_O_2_ (ODTD) 12‐oxo‐4,8‐dimethyl‐trideca‐4,8‐diene‐1‐al	236.178	237.185	259.167	291.193	2.9
2	C_20_H_32_O_2_	304.240	305.248	327.230	359.256	6.8
3	C_25_H_40_O_2_	372.303	373.310	395.292	427.318	9.6
4	C_30_H_48_O_2_	440.365	441.373	463.355	495.381	10.5
5	C_35_H_56_O_2_	508.428	509.435	531.417	563.444	10.3
6	C_40_H_64_O_2_	576.491	577.498	599.480	631.506	10.4
7	C_45_H_72_O_2_	644.553	645.561	667.543	699.569	12.4
8	C_50_H_80_O_2_	712.616	713.623	735.605	767.631	10.1
9	C_55_H_88_O_2_	780.678	781.686	803.668	835.694	8.7
10	C_60_H_96_O_2_	848.741	849.748	871.730	903.757	9.5
11	C_65_H_104_O_2_	916.804	917.811	939.793	971.819	9.0

Polyisoprene latex was treated with purified rubber oxygenase (Lcp_K30_), and cleavage products were extracted with ethylacetate and dissolved in methanol. Products were analysed by HPLC‐ESI‐MS analysis before and after purification of individual peaks by FPLC (Fig. 4). For each compound the theoretical *m/z* values and the values corresponding to the protonated ([M+H]^+^), the sodium ion adduct ([M+Na]^+^) and for the sodium ion+ methanol adduct forms ([M+Na+CH_3_OH]^+^) are indicated. The relative amounts (in %) of each prepared oligo‐isoprenoid are also provided.

## Conclusions and outlook

Polyisoprene in form of natural rubber latex is a cheap bulk compound and is available in the ton‐scale. Cleavage of polyisoprene by rubber oxygenases and separation of produced oligo‐isoprenoids is fairly possible. In this study, eleven oligo‐isoprenoids of the ‘ODTD‐family’ with one to eleven central isoprene units (*n*) between the terminal aldehyde and keto functional groups could be separately prepared. The highest yields were obtained for ODTD (RoxA_Xsp_ alone) and for the C_30_ to C_50_ compounds (Lcp_K30_ or RoxB_Xsp_ alone). Purification of oligo‐isoprenoids by FPLC can be easily up‐scaled for the mass production of oligo‐isoprenoids. The use of tyres and other materials containing vulcanized rubbers as substrates for enzymatic degradation by different rubber oxygenases is also possible; however, the presence of sulfur bridges and other components complicates the efficiency of enzymatic cleavage of vulcanized rubber waste and therefore limit – at present – the use of rubber oxygenases to the cleavage of unprocessed natural rubber latex. Mechanical, chemical and/or physical pre‐treatments of rubber wastes (e.g. grinding, solvent extraction, desulphurization) might help to make processed rubber wastes also accessible for enzymatic cleavage. Isoprenoids derived from rubber can be used for the production of fragrances, hormones and pharmaceuticals, creating interest in cheap synthesis pathways see (Förster‐Fromme and Jendrossek, 2010; Schewe *et al*., 2015). Furthermore, they can be also used in chemical or enzymatic cyclization reactions (Siedenburg *et al*., 2012, 2013) for the production of cyclic compounds or can be used as biofuels (Mewalal *et al*., 2017). This study provides purified, reactive oligo‐isoprenoids that can likely be used to convert rubber waste, e.g., from tires into precursors for the synthesis of value‐added compounds. The reactivity of the aldehydes might be directly used to form covalent bonds with other molecules (e.g. with amines). Alternatively, the keto groups of the oligo‐isoprenoids can be chemically or enzymatically reduced to the corresponding mono‐ or di‐alcohols. The reduction in the C_15_ compound ODTD to the corresponding alcohol by enzymatic reduction has been previously demonstrated (Braaz *et al*., 2005). Enzymatic generation of isoprenoid‐diols can help to provide precursors for the production of polymers from sustainably produced monomers, e.g., for the production of polyurethanes and might be an alternative to chemical methods for the conversion of polyisoprenes to polyurethanes (Anancharoenwong, 2011).

## Conflict of Interest

None declared.
